# Streptozotocin-induced β-cell damage, high fat diet, and metformin administration regulate *Hes3* expression in the adult mouse brain

**DOI:** 10.1038/s41598-018-29434-2

**Published:** 2018-07-27

**Authors:** Polyxeni Nikolakopoulou, Antonios Chatzigeorgiou, Ioannis Kourtzelis, Louiza Toutouna, Jimmy Masjkur, Carina Arps-Forker, Steven W. Poser, Jan Rozman, Birgit Rathkolb, Juan Antonio Aguilar-Pimentel, German Mouse Clinic Consortium, Lore Becker, Thomas Klopstock, Irina Treise, Dirk H. Busch, Johannes Beckers, Kristin Moreth, Raffi Bekeredjian, Lillian Garrett, Sabine M. Hölter, Annemarie Zimprich, Wolfgang Wurst, Robert Brommage, Oana Amarie, Jochen Graw, Julia Calzada-Wack, Frauke Neff, Andreas Zimmer, Manuela Östereicher, Ralph Steinkamp, Christoph Lengger, Holger Maier, Claudia Stoeger, Stefanie Leuchtenberger, Eckhard Wolf, Martin Klingenspor, Markus Ollert, Carsten Schmidt-Weber, Helmut Fuchs, Valerie Gailus-Durner, Martin Hrabe de Angelis, Vasiliki Tsata, Laura Sebastian Monasor, Maria Troullinaki, Anke Witt, Vivian Anastasiou, George Chrousos, Chun-Xia Yi, Cristina García-Cáceres, Matthias H. Tschöp, Stefan R. Bornstein, Andreas Androutsellis-Theotokis

**Affiliations:** 1 Department of Medicine, Technische Universität Dresden, Dresden, Germany; 2 Department of Clinical Pathobiochemistry, Institute for Clinical Chemistry and Laboratory Medicine, Dresden, Germany; 3 German Mouse Clinic, Institute of Experimental Genetics, Helmholtz Zentrum München, Ingolstädter Landstr. 1, 85764 Neuherberg, Germany; 4 German Center for Diabetes Research (DZD), Ingolstädter Landstr. 1, 85764 Neuherberg, Germany; 5 Institute of Molecular Animal Breeding and Biotechnology, Gene Center, Ludwig-Maximilians-University Munich, Feodor-Lynen Str. 25, 81377 Munich, Germany; 6 Chair of Molecular Nutritional Medicine, Technical University Munich, EKFZ – Else Kröner Fresenius Center for Nutritional Medicine, Gregor-Mendel-Str. 2, 85350 Freising-Weihenstephan, Germany; 7 ZIEL – Institute for Food and Health, Technical University Munich, Gregor-Mendel-Str. 2, 85350 Freising-Weihenstephan, Germany; 8 Department of Infection and Immunity, Luxembourg Institute of Health, Esch-sur-Alzette, Luxembourg; 9 Department of Dermatology and Allergy Center, Odense Research Center for Anaphylaxis, University of Southern Denmark, Odense, Denmark; 10 Center of Allergy & Environment (ZAUM), Technische Universität München, and Helmholtz Zentrum München, Ingolstädter Landstr.1, 85764 Neuherberg, Germany; 11 Chair of Experimental Genetics, School of Life Science Weihenstephan, Technische Universität München, Alte Akademie 8, 85354 Freising, Germany; 12 DFG-Center for Regenerative Therapies Dresden, Cluster of Excellence, Technische Universität Dresden, Dresden, Germany; 13 DZD/Paul Langerhans Institute Dresden of Helmholtz Centre Munich, Faculty of Medicine, Technische Universität Dresden, Dresden, Germany; 14 First Department of Pediatrics, National and Kapodistrian University of Athens Medical School, Athens, Greece; 15 Aghia Sophia Children’s Hospital, Athens, Greece; 16 Department of Endocrinology and Metabolism, Academic Medical Center, University of Amsterdam, Amsterdam, The Netherlands; 17 Helmholtz Diabetes Center (HDC) & German Center for Diabetes Research (DZD), Helmholtz Zentrum München, 85764 Neuherberg, Germany; 18 Division of Metabolic Diseases, Technische Universität München, 80333 Munich, Germany; 19 Division of Cancer and Stem Cells, University of Nottingham, Nottingham, NG7 2RD UK; 20 Department of Neurology, Friedrich-Baur-Institut, Ludwig-Maximilians-Universität München, Ziemssenstrasse 1a, 80336 Munich, Germany; 21 Deutsches Institut für Neurodegenerative Erkrankungen (DZNE) Site Munich, Schillerstrasse 44, 80336 Munich, Germany; 22 Munich Cluster for Systems Neurology (SyNergy), Adolf-Butenandt-Institut, Ludwig-Maximilians-Universität München, Schillerstrasse 44, 80336 Munich, Germany; 23 German Network for Mitochondrial Disorders (mitoNET), Munich, Germany; 24 German Center for Vertigo and Balance Disorders, Munich, Germany; 25 Institute for Medical Microbiology, Immunology and Hygiene, Technical University of Munich, Trogerstrasse 30, 81675 Munich, Germany; 26 Chair of Experimental Genetics, Center of Life and Food Sciences Weihenstephan, Technische Universität München, Ingolstaedter Landstrasse 1, 85354 Freising-Weihenstephan, Germany; 27 Member of German Center for Diabetes Research (DZD), Ingolstaedter Landstraße 1, 85764 Neuherberg, Germany; 28 Department of Cardiology, University of Heidelberg, Im Neuenheimer Feld 410, 69120 Heidelberg, Germany; 29 Institute of Developmental Genetics, Helmholtz Zentrum München, German Research Center for Environmental Health GmbH, Ingolstaedter Landstrasse 1, 85764 Neuherberg, Germany; 30 Chair of Developmental Genetics, Center of Life and Food Sciences Weihenstephan, Technische Universität München, Ingolstaedter Landstrasse 1, 85764 Neuherberg, Germany; 31 Institute of Pathology, Helmholtz Zentrum München, German Research Center for Environmental Health GmbH, Ingolstaedter Landstrasse 1, 85764 Neuherberg, Germany; 32 Institute of Molecular Psychiatry, Medical Faculty, University of Bonn, Sigmund-Freud-Strasse 25, 53127 Bonn, Germany

## Abstract

Diabetes mellitus is a group of disorders characterized by prolonged high levels of circulating blood glucose. Type 1 diabetes is caused by decreased insulin production in the pancreas whereas type 2 diabetes may develop due to obesity and lack of exercise; it begins with insulin resistance whereby cells fail to respond properly to insulin and it may also progress to decreased insulin levels. The brain is an important target for insulin, and there is great interest in understanding how diabetes affects the brain. In addition to the direct effects of insulin on the brain, diabetes may also impact the brain through modulation of the inflammatory system. Here we investigate how perturbation of circulating insulin levels affects the expression of *Hes3*, a transcription factor expressed in neural stem and progenitor cells that is involved in tissue regeneration. Our data show that streptozotocin-induced β-cell damage, high fat diet, as well as metformin, a common type 2 diabetes medication, regulate *Hes3* levels in the brain. This work suggests that *Hes3* is a valuable biomarker helping to monitor the state of endogenous neural stem and progenitor cells in the context of diabetes mellitus.

## Introduction

There is concern that diabetes and, more generally, aberrant insulin regulation, have a negative impact on brain function^1^. In fact, even common medication for diabetes such as metformin may impact the brain in ways that are not yet well understood as metformin has been shown to alter the self-renewal and differentiation properties of neural stem cells *in vitro* and *in vivo*^2,3^. It is, therefore, important to identify molecular mechanisms by which diabetes mellitus may affect the brain, either directly via alterations in the levels of circulating glucose and insulin or indirectly through effects on the immune/inflammatory system. Here we investigated how streptozotocin-induced β-cell damage, high fat diet, and metformin administration regulate the expression of *Hairy and Enhancer of Split 3* (*Hes3*) in the adult mouse brain.

We focused on *Hes3* because (a) we previously showed that it is involved in various paradigms of brain damage and regeneration^4–7^, (b) it is also expressed in neural stem cells^4^, and (c) *Hes3* is regulated by insulin^4–6^. *Hes3* belongs to the Hes superfamily of basic helix-loop-helix (bHLH) transcription factors that include the Hes and Hey (Hes-related with YRPW motif) members^8–10^. *Hes1* and *Hes5* are direct targets of Notch signaling and their expression is often used as an indicator of canonical Notch signaling activity^10^. In contrast, *Hes3* is an indirect target of Notch signaling; following Notch receptor activation, a pathway involving phosphatidylinositol-4, 5-bisphosphate 3 (PI3) kinase, Protein kinase B (Akt), mechanistic target of rapamycin (mTOR), and Signal transducer and activator of transcription 3 - Serine (STAT3-Ser) phosphorylation leads to *Hes3* expression, which can be used as an indicator of the activity of this non-canonical Notch signaling branch^4^.

*Hes3* is of interest because emerging data shows that it is an important regulator of regeneration in both the pancreas and brain. In cultured mouse insulinoma cells (MIN6), *Hes3* knockdown and overexpression studies revealed that *Hes3* regulates the expression of pancreatic and duodenal homeobox 1 (Pdx1), an important gene in pancreatic islet health and insulin production; it also regulates the expression of insulin itself^11^. *Hes3* null mice are more sensitive to pancreatic islet damage by the toxin streptozotocin (STZ; used to model type 1 diabetes), compared to wild type (WT) mice, and regenerate beta cell mass less efficiently^11,12^. In the brain, *Hes3* is expressed in putative neural stem cells (NSCs) and progenitor cells^4,6^. Cultured NSCs also express *Hes3*; expression is lost following their differentiation^13–15^. Various pharmacological treatments that induce *Hes3* expression promote cell survival in culture and the number of *Hes3*-expressing cells *in vivo*^4–6,13–17^. This is followed by powerful neuroprotection and disease modification in models of ischemic stroke and Parkinson’s disease^4–6^. *Hes3* null mice exhibit lower levels of myelin basic protein (MBP) in the brain, indicating insufficient numbers of oligodendrocytes or reduced myelination^7^. In summary, *Hes3* plays important roles in various tissues and organs, including the brain, where it protects them from damage and enables them to regenerate efficiently.

In this work we demonstrate, for the first time, that the expression of *Hes3* in the brain is regulated in mice subjected to streptozotocin-induced β-cell damage, high fat diet, and metformin administration. We establish *Hes3* as a biomarker to monitor the brain in animal models that are widely used to study various aspects of diabetes mellitus. Future studies will address whether *Hes3* is also regulated in diabetes patients, which parameters of insulin deregulation and/or diabetes mellitus are primarily responsible for *Hes3* regulation, and the roles that *Hes3* plays in the progression of diabetes-related phenotypes.

## Results

### Streptozotocin-induced β–cell damage and high fat diet regulate *Hes3* expression in the brain

As described in the introduction, we hypothesized that brain *Hes3* expression would be altered in mouse models of diabetes, in which insulin signaling is perturbed. Such a result would provide novel information, at the molecular level, of how such perturbations might be affecting the brain. We used streptozotocin (STZ) to induce insulin deficiency. STZ-induced β–cell damage is an established model to study type 1 diabetes in rodents. High dose STZ induces hyperglycemia and leads to insulin deficiency resulting from selective β-cell damage in the pancreas^18,19^. Furthermore, we studied mice fed a high-fat diet (HFD) as there is a general agreement that feeding a high calorie diet results in impaired glucose homeostasis and at least a pre-diabetic state comprising hyperglycemia, hyperinsulinemia and insulin resistance^20^.

To measure *Hes3* expression, we prepared mRNA extracts from mouse brains where the olfactory bulb and all parts caudal to the cortex were removed. Mice were carefully age-matched because, as we observed using PCR analysis, expression of *Hes3* (both isoforms: *Hes3a* and *Hes3b*) drops with age (Fig. S1a). A polyclonal antibody against *Hes3* further confirmed the reduction in *Hes3* expression with age (Fig. S1b,c). The data are consistent with a role of *Hes3* in the NSC/progenitor cell population.

**Figure 1:**
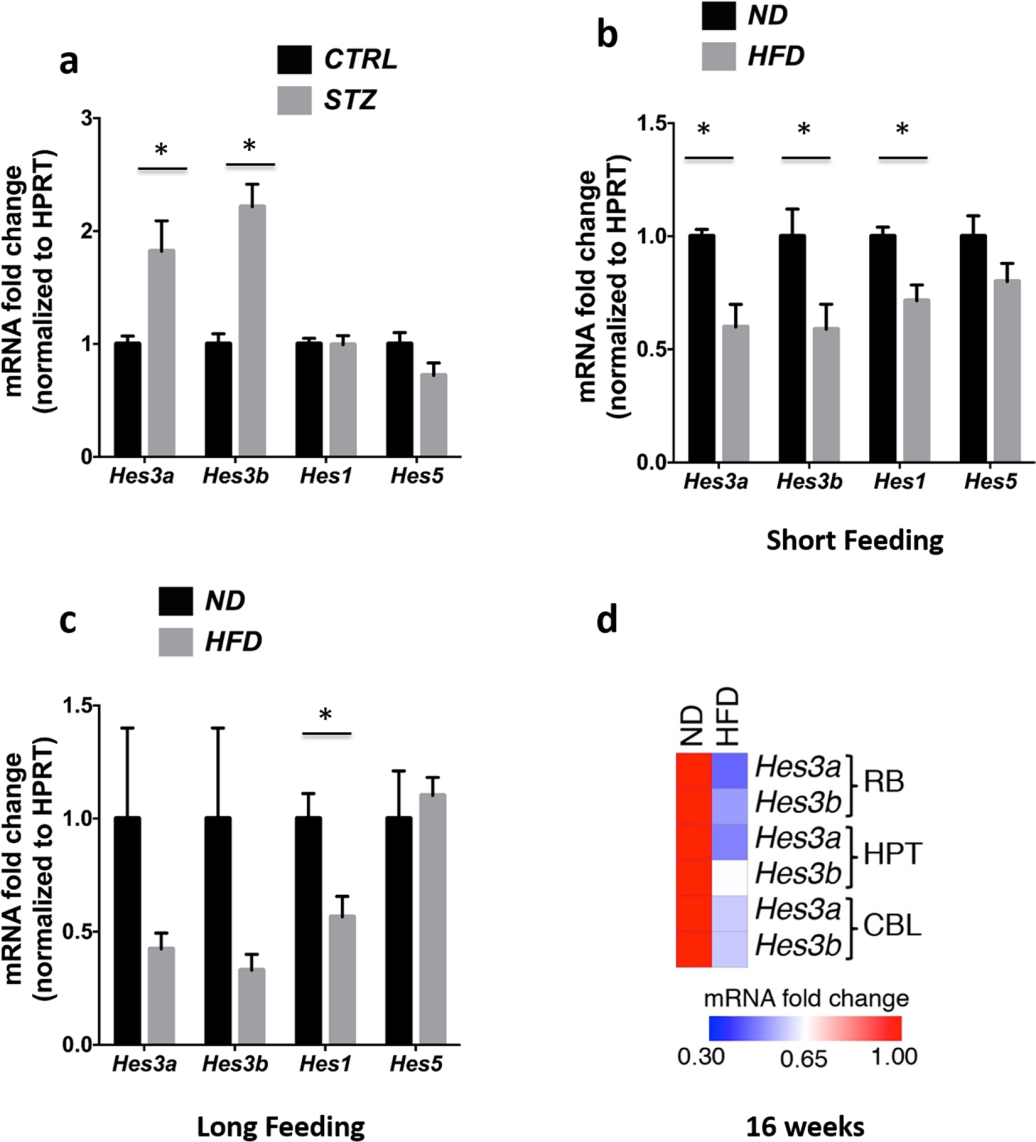
Streptozotocin-induced β–cell damage and high fat diet regulate *Hes3* expression in the brain. (**a**) STZ regulates *Hes3*a and *Hes3*b levels (N = 6–7). (**b**,**c**) HFD (short and long feeding) regulates Hes/Hey gene expression (N = 4–7). (**d**) HFD (16 weeks) regulates *Hes3* expression in different brain areas. The heatmap shows average gene expression for *Hes3a* and *Hes3b* in different brain areas (BR, HPT, CBL; N = 5–8). [Data are means ± SEM. Mann-Whitney test; *p < 0.05. HPRT was used as a reference gene]. See also Fig. S1.

In the pancreas, the toxin streptozotocin (STZ) induces a powerful increase in *Hes3* expression, possibly in an effort to promote regeneration of pancreatic islet cells^11,12,21^. Here we addressed whether similar effects can also be observed in the brain. STZ is used to damage pancreatic islet cells and produce animal models for the study of type 1 diabetes that exhibit reduced production and systemic circulation of insulin^18,19,22^. Consistent with published studies, mice treated with STZ exhibited increased glucose levels and reduced insulin levels (Fig. S1d,e). In these mice, *Hes3a* and *Hes3b* mRNA levels in the brain were significantly increased; in contrast, the mRNA levels of the canonical Notch signaling targets *Hes1* and *Hes5* were not significantly altered (Fig. 1a). These data show that intraperitoneal administration of STZ leads to *Hes3* expression changes in the brain.

HFD in mice leads to a complex condition that involves elevated circulating insulin (an activator of *Hes3*) as well as complex inflammatory responses (some of which promote and others that are predicted to oppose *Hes3* expression)^11,15^. It is therefore difficult to predict the effect of particular HFD paradigms on brain *Hes3*; here we investigated this question using established HFD protocols. In both the short and long HFD feeding groups the mice gained BW and exhibited increased insulin and glucose levels (Fig. S1f–h). In the short HFD feeding group, *Hes3a, Hes3b, Hes1, but not Hes5* expression were reduced, relative to the short normal diet (ND) feeding group; in the long HFD feeding group, *Hes3a* and *Hes3b* expression showed a tendency to decrease, compared to the long ND feeding group, but did not reach statistical significance. Again, *Hes1* but not *Hes5* levels also dropped (Fig. 1b,c). We used a separate group of mice, fed a HFD for an intermediate period (16 weeks), to identify particular brain areas where *Hes3* mRNA levels are regulated. With this group, we dissected distinct brain areas: Hypothalamus (HPT), Cerebellum (CBL), and the Remaining Brain without HPT, CBL, and olfactory bulbs that we denote here as “RB”. HPT is involved in metabolic regulation and it expresses *Hes3*^23^. *Hes3* is highly expressed in the CBL although its roles are unknown; RB would provide an overview of *Hes3* regulation in the remaining brain areas. In this group of mice we measured significant reductions in both *Hes3*a and *Hes3*b levels in RB, HPT, and CBL (Significant changes: RB: *Hes3a* and *Hes3b*; HPT: *Hes3a*; CBL: *Hes3a* and *Hes3b*) (Fig. 1d). These results show that streptozotocin-induced β-cell damage and HFD induce significant alterations in the expression of *Hes3* in the brain.

### Metformin regulates *Hes3* expression *in vivo* and *in vitro*

We hypothesized that metformin administration would alter brain *Hes3* expression. To show this could have important clinical connotations as many patients are prescribed metformin. Metformin is a widely prescribed medication for type 2 and some cases of type 1 diabetes mellitus that opposes hyperglycemia and improves insulin sensitivity^24,25^. It regulates a variety of signaling pathways, suggesting that it may have additional effects. Indeed, metformin also appears to have anti-inflammatory effects irrespective of diabetes mellitus status^26,27^, anti-tumor effects^28,29^, as well as several effects in the living brain including the promotion of neurogenesis and improved spatial memory formation^30,31^. Because it regulates signaling pathways that intercept with *Hes3*, such as mTOR^4,32,33^, we addressed the effect of metformin on *Hes3* expression in the living brain.

**Figure 2:**
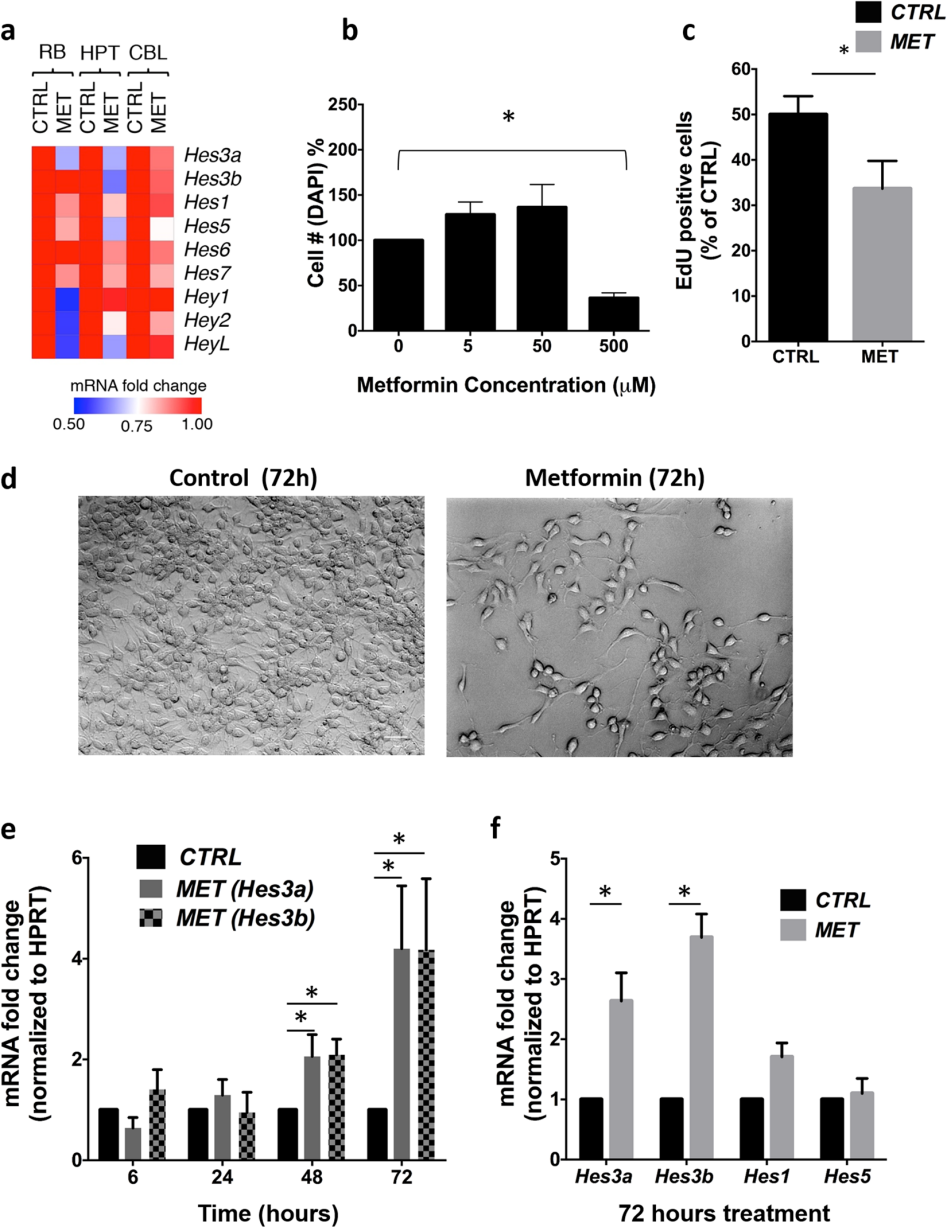
Metformin regulates *Hes3* expression *in vivo* and *in vitro*. (**a**) Metformin in drinking water for 2 months regulates Hes/Hey gene mRNA levels in the brain (N = 5–12; CTRL: Control; MET: Metformin). The heatmap shows average gene expression levels in different areas of the brain. (**b**) Metformin reduces cell number (DAPI-stained nuclei counts) in a dose-dependent manner (N = 4; 72 h, 500 µM). (**c**) Metformin (500 µM) reduces EdU incorporation *in vitro* (N = 3; 72 h; 1-tailed t-test). (**d**) Brightfield images of control and metformin-treated (500 µM, 72 hours) primary fNSC cultures. [Scale bar: 30 μm]. (**e**,**f**) Metformin (500 µM) regulates *Hes3a* and *Hes3b* mRNA levels *in vitro* (N = 3, 72 h). [Data are means ± SEM. Mann-Whitney test unless noted otherwise; *p < 0.05. HPRT was used as a reference gene; for the *in vitro* experiments, data were collected from at least 3 separate experiments]. See also Fig. S2.

We isolated RNA from RB, HPT, and CBL as described above. Metformin affected the expression of multiple Hes/Hey genes in the living brain (Significant changes: RB: *Hes6*, *Hey1*, *Hey2*, *HeyL*; HPT: *Hes3b*, *Hes5*, *Hey2*, *HeyL*; CBL: No significant changes) (Figs 2a; S2a–d). Metformin did not significantly affect BW; fasting glucose levels were slightly but significantly reduced; fasting insulin levels were slightly but not significantly increased (Fig. S2e–h). Overall, metformin reduced *Hes3* expression in the hypothalamus of the adult mouse brain.

To address whether metformin acts directly on NSCs, we used established culture systems of primary mouse fetal NSCs (fNSCs). Dose response experiments demonstrated that 500 µM metformin reduced cell number and 5-ethynyl-2′-deoxyuridine (EdU) incorporation; cell morphology changed to a more differentiated appearance with longer processes (Figs 2b–d; S2i). Together, these results suggest that metformin opposes self-renewal and promotes cell differentiation. Time course experiments revealed a time-dependent increase in *Hes3*a and *Hes3*b mRNA levels; at 72 hours we observed increased mRNA levels of *Hes3*a and *Hes3*b but not *Hes1* or *Hes5* (Fig. 2e,f).

These data show that metformin increases *Hes3* levels in cultured NSCs, although it decreases *Hes3* expression *in vivo*. We speculate that the expression increase *in vitro* may be a response to the stress caused to the cells by metformin or due to changes induced by the onset of the differentiation process. In support of this, we show a transient increase in immunoreactivity for *Hes3* when cultured NSCs start expressing markers of neurons (TUJ1) and oligodendrocytes (CNPase) (Fig. S2j). Overall, our data show effects of metformin on *Hes3* expression in NSCs isolated from the fetal mouse brain. Future experiments may address whether the changes in *Hes3* expression mediate effects of metformin to brain function.

Exendin-4 (Ex-4) is another commonly used type-2 diabetes medication. We previously showed that it induces *Hes3* expression in a cultured mouse insulinoma cell line^11^. Unlike metformin, Ex-4 promoted cultured NSC growth at concentrations (200 nM) that induced *Hes3* expression (Fig. S3a,b). Also unlike metformin, Ex-4 did not reduce EdU incorporation (Fig. S3c,d). qPCR analysis showed that Ex-4 significantly increased *Hes3a* and *Hes3b* mRNA levels, in a time-dependent manner (Fig. S3e). At 72 h of treatment (the time point when *Hes3* expression induction reached significance), we did not observe a significant change in *Hes1* expression; however, we did measure a significant induction of *Hes5* expression (Fig. S3f). These results dissociate the increase in *Hes3* expression from cell growth and are consistent with the hypothesis that the increase in *Hes3* expression by metformin may be part of a stress response to the treatment.

### *Hes3* null mice exhibit a quasi-normal phenotype

We established *Hes3* expression as an indicator that streptozotocin-induced β-cell damage, high fat diet, and metformin administration affect the brain. It is not yet clear what consequences these changes may have in the health of the experimental models, despite the fact that we previously showed, using *Hes3* null mice, that the complete lack of *Hes3* leads to increased sensitivity and impaired regeneration in the pancreas^11,12^ (using the STZ model) and in the brain^7^ (following cuprizone-induced damage to oligodendrocytes). Extensive future work with conditional genetic mouse models may address the roles of *Hes3* in different tissues and organs, in the progression of diabetic and brain-related phenotypes. However, in order to obtain first-level information on possible *Hes3* roles, we performed an extensive phenotypic analysis of the *Hes3* null (“knockout”) mouse strain.

Homozygous *Hes3* null mice are generally healthy and breed normally^9^ but also have phenotypes that become obvious under stress^5,7,11,12^. We performed particular phenotypic analyses of the *Hes3* null mice following either a ND or a HFD. A summary of the results is presented in Table 1. The full report is provided at https://www.mouseclinic.de (Click on “phenomap” and search for project “*Hes3*_KO”).

**Table 1:** Summary of phenotypic analysis.

Screen	Method	Phenotype summary Hes 3 null
**NORMAL DIET**		
Energy Metabolism	Indirect calorimetry NMR	-None -Trend towards increased fat content over time mainly in male mutants
Behavior	Open field	-None
	Acoustic startle response, PPI	-None
Neurology	Modified SHIRPA, Auditory brain stem response Rotarod Grip strength	-None -None -None -Small trend towards increased fore paw force
Nociception	Hot plate	- Shorter reaction time for the first pain reaction in females, trend towards hyperalgesia
Dysmorphology	Anatomical observation, X-ray, MicroCT scans (dissected bones)	-None
Cardiovascular	Awake ECG Echocardiography	-No clear phenotype -Very mild increase in septum width in systole (males). -Very mild reduction in heart rate and thus RR interval prolongation -Very mild alterations in QRS, ST and Qtdisp intervals (probably by chance)
Eye	Scheimpflug imaging, OCT, LIB, drum	-Slight decrease in retinal thickness (females)
Clinical Chemistry	IpGTT Insulin levels Clinical chemical analysis Hematology	-IPGTT: Mild trend downwards in males and upwards in females for AUC values. -None -Trend towards changes in creatinine and fructosamine (mainly females) concentrations -Hematology: Slightly lower platelet distribution width
Immunology	Flow cytometry analysis of Peripheral Blood Leukocytes	-Subtle alterations in the leukocyte subpopulations, however no evidence for pathological effects in the immune system: -increased frequency of B cells -decreased frequency of CD4 single positive T cells -increased frequency of CD4 CD8 double positive T cells -increased proportion of CD8 single positive T cells (females) -increased CD44 expression on CD4^+^ T cells
Allergy	ELISA (IgE concentration) TEWL	-None -None
Pathology	Macro & microscopic analysis	-None
**HIGH FAT DIET**		
Energy Metabolism	Indirect calorimetry, NMR	-None
	Body temperature	-Slight increase in males and females
	Fat mass	-Mild decrease in females
Allergy	TEWL	-Slight increase in females
	Body surface temperature	-None
Clinical Chemistry	IpGTT	-IPGTT: Mild trend downwards in males for AUC values.

WT and *Hes3* null mice were subjected to a number of phenotypic assays, as summarized in the table. A full report of the analyses is presented in the Supplementary Material section. [NMR: Nuclear magnetic Resonance; PPI: Prepulse Inhibition; SHIRPA: http://www.har.mrc.ac.uk/services/phenotyping/neurology/shirpa.html; MicroCT: Micro Computer Tomography; ECG: electrocardiography; OCT: Optical Coherence Tomography; LIB: laser interference biometry; ipGTT: intraperitoneal glucose tolerance test; PBCs: Peripheral Blood Leukocytes; ELISA: enzyme-linked immunosorbent assay; TEWL: Transepidermal water loss; AUC: Area Under Curve].

First, we investigated whether the expression of other members of the Hes/Hey gene family is altered in the brains of the *Hes3* null mice. We expected this because members of this family are known to suppress the expression of other members^10^. Because *Hes1* and *Hes5* are established mediators of canonical Notch signaling, such a result would demonstrate that key pathways in neural stem cell biology are also affected.

**Figure 3:**
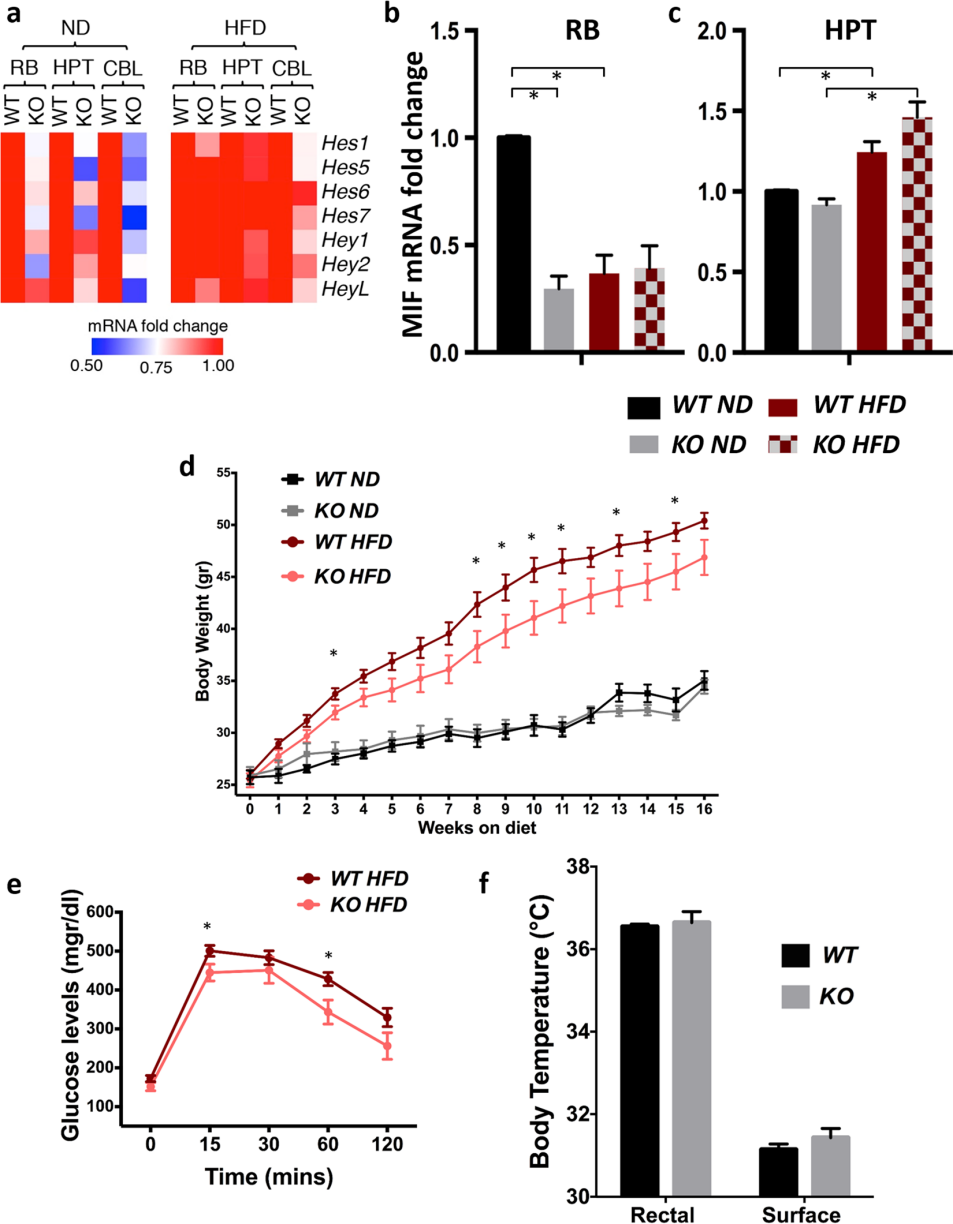
*Hes3* null mice exhibit a quasi-normal phenotype. (**a**) Heatmap of the average qPCR gene expression levels of different Hes/Hey genes in WT and *Hes3* null mice in the brain areas indicated under ND and under HFD conditions (for 16 weeks; N = 4–8). (**b**) MIF expression in RB of WT and *Hes3* null mice under ND and HFD (qPCR; N = 3–6). (**c**) MIF expression in HPT of WT and *Hes3* null mice under ND and HFD (qPCR; N = 4–7). (**d**) BW progression in mice fed a ND (for 16 weeks; N = 5, 5, same cohort as in (**a**)) or a HFD (for 16 weeks; N = 11, 9). (**e**) *Hes3* null mice exhibit lower scores (and AUC values) in the ipGTT assay in HFD (after 24 weeks of HFD; see https://www.mouseclinic.de) (N = 11,9). (**f**) *Hes3* null mice exhibit a trend towards higher rectal and body surface temperature than controls [Same cohort of mice as in (**e**); N = 7,7]. [Data are means ± SEM. Mann-Whitney test; *p < 0.05. HPRT was used as a reference gene]. See also Fig. S4.

We found that in ND, male *Hes3* null mice showed significant alterations in the expression of other Hes/Hey gene family members in the brain, compared to WT; these alterations were much less pronounced in HFD (Figs 3a; S4a–g). These results show that the lack of *Hes3* alters the equilibrium of the other Hes/Hey genes and that this outcome is affected by diet.

We continued our investigation of the consequences of lack of *Hes3* by focusing on the expression of Microphage Migration Inhibitory Factor (*MIF*). Low-grade inflammation is a hallmark of type 2 diabetes mellitus. Of the inflammatory cytokines, *MIF* has been demonstrated to positively regulate *Hes3* expression in isolated NSC cultures^15^. We addressed how MIF levels are altered by diet and by the lack of *Hes3* (comparing WT and *Hes3* null mice).

We compared *MIF* mRNA levels in RB and HPT of WT and *Hes3* null mice in both ND and HFD (Fig. 3b,c). In ND we observed the following: *Hes3* null mice express a lower amount of *MIF* in the RB compared to WT. In HPT, the difference did not reach statistical significance. In HFD we observed the following: MIF expression showed no significant difference between WT and *Hes3* null mice in the RB, whereas in the HPT there was a non-statistically significant trend towards an increase. MIF expression level in WT mice was affected by diet; HFD reduced MIF expression in the RB. In contrast, we observed an increase in the HPT.

Whereas MIF levels were altered by diet in WT mice in both the RB and HPT, *Hes3* null mice did not exhibit changes in MIF expression in the RB due to diet. They did, however, exhibit an increase in HPT, similarly to WT mice. The results suggest a complex interplay among *Hes3*, diet, and MIF in different brain areas and call for additional studies to address the potential reciprocal regulation between *Hes3* and the inflammatory system.

The expression changes in the brain prompted us to investigate potential phenotypes that relate to brain function. We performed a set of neurological phenotypic analyses in *Hes3* null and WT mice fed a ND. We did not observe significant differences between the *Hes3* null and WT mice in terms of behavior (open field test), neurology (Modified SHIRPA, auditory brain stem response, rotarod test, grip strength), nociception (hot plate test), and eye functions (Scheimpflug imaging, OCT, LIB, drum). Regarding energy metabolism, indirect calorimetry showed no differences in ND, whereas NMR showed a trend towards increased fat content over time mainly in male mutants. We also found no dysmorphology phenotypes (Anatomical observation, X-ray, MicroCT scans of dissected bones), in ND. We also did not find any clear cardiovascular phenotypes in ND; awake ECG Echocardiography in ND showed a very mild increase in septum width in systole (males), a very mild reduction in heart rate and very mild alterations in QRS, ST and Qtdisp intervals (probably by chance) (Table 1; https://www.mouseclinic.de; Fig. S5a-l, and Supplementary Material-GMC Report).

The very mild phenotypes relating to behavior, neurology, and nociception pushed our focus to other potential phenotypes, for example, those that are relevant to metabolism. Using male mice, we found that in ND, *Hes3* null mice behaved similarly to wild-type (WT) mice in terms of BW gain; in HFD, *Hes3* null mice gained less BW, compared to WT mice (Fig. 3d). In both ND and HFD, *Hes3* null mice showed lower glucose levels during the course of the ipGTT compared to WT mice, resulting in slightly lower AUC values especially for the second part of the test [AUC (30–120 mins)], which might indicate an improved glucose tolerance (HFD: Fig. 3e; ND: Supplementary Material-GMC Report). Male *Hes3* null mice fed a HFD also exhibited a trend (p = 0.053) towards higher rectal and body surface temperature (Fig. 3f). [Two Way ANOVA analysis revealed a significant increase in the body temperature of WT versus KO mice when both male and female mice were analyzed together (p = 0.036; N = 14,14)] (Supplementary Material-GMC Report).

Beyond the brain, *Hes3* null mice in ND also exhibited subtle alterations in the leukocyte subpopulations [increased frequency of B cells, decreased frequency of CD4 single positive T cells, increased frequency of CD4 CD8 double positive T cells, increased proportion of CD8 single positive T cells (females), and increased CD44 expression on CD4+ T cells], although no evidence for pathological effects in the immune system was observed (Table 1 and Supplementary Material-GMC Report).

Overall, the phenotypic analyses of the *Hes3* null mouse strain show mild phenotypes along with an altered equilibrium of the expression of multiple other Hes/Hey genes in the brain and point towards multiple roles that may be addressed in future studies.

## Discussion

Diabetes affects the brain in ways that are insufficiently understood^34^. Ironically, diabetes medication such as metformin also affects the brain, and we don’t know the consequences to the patient. It is important to reveal the molecular mechanisms that are affected in order to be able to predict outcome and design appropriate therapeutic interventions. A sensitive biomarker in the brain whose expression levels change in response to disease progression and metformin administration would be a valuable new tool. Such a biomarker would be even more valuable if it is already known to play roles in brain regeneration.

Here we used a mouse model of type 1 diabetes mellitus (the streptozotocin-induced β-cell damage model), a mouse model of high fat diet exhibiting systemic inflammation and insulin resistance, often used to study aspects of type 2 diabetes, and a mouse model of metformin administration to establish the transcription factor *Hes3* as such a biomarker. We show that the expression of *Hes3* in the brain is regulated in all three mouse models. We present a conceptual diagram of how *Hes3* may be regulated by a number of parameters, including signal transduction pathways, inflammatory responses, insulin levels, age, etc. (Fig. S6).

Future work may address the mechanisms by which *Hes3* is regulated in the animal models we employed, as well as, potentially in patients with type 1 and type 2 diabetes, and during metformin administration. It may also address the impact that Hes3 expression changes might have to the brain. For now, we may speculate on a number of possibilities.

First, insulin may directly regulate *Hes3*+ cells in the brain. Type 1 and type 2 diabetes mellitus as well as being overweight or obese are known risk factors for developing cognitive impairment and dementia^35–40^. In part, this risk is thought to be due to aberrant insulin actions directly to the brain. Beyond stimulating glucose metabolism, insulin (along with the related hormone Insulin-like growth factor 1, IGF-1) has multiple functions in the brain, supporting the survival of neurons and oligodendrocytes^41^, promoting synaptic integrity and plasticity^42^, and helping working memory and cognition^43^. At the signal transduction level, post-mortem analysis of brains from patients with Alzheimer’s disease reveals perturbed signaling downstream of the insulin receptor, including reduced insulin and IGF-1 binding to their receptors and impaired PI3K/Akt signaling^44–47^. *Hes3* is regulated by insulin, as we previously showed in neural stem cell cultures as well as *in vivo*^4–6^. In fact, *Hes3* is a key component of a signal transduction pathway that is involved in a variety of regeneration paradigms in different cell types *in vitro* and in different tissues *in vivo*^4–6,11,12,14,15,21,48–50^. It is possible, therefore, that altering circulating insulin levels may lead to sufficient changes in brain interstitial fluid levels, and that may directly affect *Hes3*-expressing cells. But it has proven complicated to determine how insulin levels in the interstitial fluid of the brain change when circulating insulin levels are altered^51–53^. Cerebrospinal fluid (CSF) levels have been used as surrogate measurements for brain interstitial levels. However, this may not be an accurate measurement as insulin is thought to predominantly enter the brain via the blood-brain barrier, as has been expertly reviewed previously^54^. In addition, elevated extracellular glucose concentrations have been suggested to be able to induce insulin production from cerebral cortical neuroglial cells^52–55^; how that may contribute to local *Hes3* expression regulation is not yet addressed. Alternatively, insulin that reaches the cerebrospinal fluid (CSF) may also be able to induce *Hes3* expression in certain brain areas adjacent to the ventricles; in support of this, in the original studies demonstrating endogenous NSC activation by insulin and other reagents, the treatments were injected into the lateral ventricles of adult rats^4–6^.

Second, oxidative and inflammatory responses may also play an important role in regulating *Hes3* expression in different cell types^56,57^. It is important to not group all inflammatory cytokines together when addressing their effects on *Hes3*. Whereas many inflammatory cytokines (e.g., the interleukin family) activate the JAK-STAT signaling pathway and would thus be expected to suppress *Hes3* expression, *MIF* has been shown to promote it via an Akt/mTOR/STAT3-Ser mechanism^11,12,15,21^. Therefore, the precise inflammatory responses activated in different disease models (or, at different stages of the same model) may contribute to the exact *Hes3* expression patterns that we observed. Such a task will require a detailed determination of the production of multiple cytokines at different time points, and an assessment of their potential to induce or suppress *Hes3* expression. A better understanding of the interaction between specific inflammatory responses and *Hes3* may help inform drug discovery programs aimed at both modulating inflammation and protecting brain tissue. Future studies may also address how *Hes3* expression is specifically altered by oxidative and inflammatory stress components such as oxygen radicals produced by the mitochondria of affected cells and activated, nuclear NF-kB. Cross-talk between NF-kB and JAK-STAT signaling as well as between reactive oxygen species and JAK-STAT increases the complexity by which *Hes3* may be regulated^58–60^.

In addition to the disease models themselves, we hypothesized that metformin, a common medication for type 2 diabetes mellitus^61,62^ would also affect brain *Hes3* levels. Our data show different effects of metformin on *Hes3* expression *in vitro* and *in vivo*, arguing for complex mechanisms by which metformin regulates *Hes3* expression. This could be due to the broad range of signaling pathways affected by metformin that include several known modulators of *Hes3*^4,63,64^. Although the precise molecular mechanisms by which metformin affects cells are not fully elucidated, studies from various cell systems have identified a number of signaling pathways that are involved. In triple-negative breast cancer cell lines, metformin was shown to oppose both JAK/STAT3-Tyr activity and STAT3-Ser phosphorylation^65^. It is possible that, depending on which of the two branches of STAT3 is mostly affected, the outcome may either favor or oppose *Hes3* expression because *Hes3* is positively regulated by inducers of STAT3-Ser phosphorylation and negatively regulated by JAK kinase which leads to STAT3-Tyr phosphorylation^4^. In addition, metformin opposes mTOR activation, via 5′ AMP-activated protein kinase (AMPK) stimulation^32,33,66–68^, a function that should also oppose *Hes3* expression (mTOR is a positive regulator of *Hes3* expression^4^). Metformin also regulates insulin-like growth factor 1 (IGF-1) and p38 mitogen-activated protein kinases (p38 MAPK) which are signaling components with prominent roles in the self-renewal of NSCs and the regulation of *Hes3* expression^4,5,64^. Inflammatory responses triggered by metformin (as well as by type 2 diabetes itself)^69–71^ further complicate the predictability of its effects on *Hes3* expression because different inflammatory cytokines may have opposite effects on *Hes3* expression (depending on whether they predominantly stimulate the JAK-STAT or Akt/mTOR pathways, for example). It will be interesting to determine whether the complex mechanisms by which metformin may regulate *Hes3* contribute to the lack of consensus regarding the drug’s potential role as a therapeutic agent in neurodegenerative disease^72,73^. It is conceivable that patients on metformin will benefit from concomitant treatments that modulate the inflammatory response in the brain such that *Hes3* levels are maintained within an appropriate range. For example, promoting *MIF* responses while suppressing interleukin responses may have beneficial effects on the neural stem/progenitor population, on neurons, and on cognitive function.

Our data implicate aging in the regulation of *Hes3*. This may not be too surprising, given that there is a general consensus that neural stem cell biomarkers decrease with age. Our observation that *Hes3* expression in the brain drops with age might have important connotations in diabetes mellitus. This is in light of studies suggesting differences between children and adults with type 1 diabetes in the way that their brain responds to the disease. In an imaging study involving a large cohort of children with type 1 diabetes, it was revealed that hippocampal volumes were increased in the children with the largest number of severe hypoglycemic episodes^74^. This result was interpreted as a manifestation of sensitivity of the hippocampus to acute hypoglycemia. It is contrary to data obtained from studies with adult type 1 diabetes patients that revealed no changes in hippocampal volume^75^. Animal studies reinforce the hypothesis that postnatal age affects the way that the brain responds to hypoglycemia^1,76^. It will be of significant interest to determine the potential role of *Hes3* in this age-related effect. It will also be valuable to determine whether the abundant, *Hes3*-expressing endogenous NSC/progenitor cell population in the hippocampus is affected in these children. The postnatal hippocampus is one of the few brain areas where regular neurogenesis is observed from a local pool of stem/progenitor cells^77^. Many putative stem/progenitor cells in the hippocampus co-express *Hes3* and their number increases *in vivo* when pharmacological agents that induce *Hes3* expression are injected into the brain^17^. Therefore, one could expect that the combination of a high-*Hes3* young brain and elevated *Hes3* expression due to diabetes mellitus could impact the stem cell-rich cytoarchitecture of the hippocampus. Given that MIF expression drops with age^78,79^ and is associated with longevity^80^, it is intriguing to speculate that age-dependent MIF expression changes may be, in part, responsible for the accompanying drop in *Hes3* expression.

The age-based differences in brain *Hes3* expression are particularly relevant in light of current efforts to assess new clinical potential of metformin. One study (Targeting Aging with Metformin, TAME; https://www.afar.org/natgeo/) will assess its anti-aging potential^81^; it may be of interest to obtain more information on how *Hes3* expression is regulated in humans by age and by metformin itself. The other study (Autoimmune Diabetes Accelerator Prevention Trial, adAPT; http://adaptdiabetes.org/) involves administering metformin to healthy children who are at risk of developing type 1 diabetes as a prevention strategy; it may be beneficial to assess differences in brain *Hes3* expression in detail between children and adults in order to potentially better predict unforeseen consequences.

Given the potential of *Hes3* as a biomarker, it is valuable to obtain information on its potential roles. In our past work and here we have focused on measuring *Hes3* expression changes in the damaged and regenerating pancreas and brain. But it is expressed in various other tissues as well, where its roles are much less known. Here we provide a first-order phenotypic analysis of *Hes3* null mice. Future studies may involve the use of conditional and inducible genetic mouse models so as to obtain phenotypic information on Hes3 in particular time points and tissues. For now, the phenotypic analysis we present provides some clues to its roles.

*Hes3* null mice exhibit differences in the expression of other Hes/Hey gene family members in the brain, relative to WT mice. They also show altered MIF expression as well as differences in leukocyte lineage frequencies are (albeit mildly), suggesting potential consequences in the levels of inflammatory cytokines and in chronic low-grade systemic inflammation, a risk factor in many patients with obesity^82^.

These differences do not seem to obviously affect brain function as phenotypic analyses on behavior, neurology, nociception, and eye function showed no significant differences between *Hes3* null and WT mice. It is possible, however, that, in the context of an appropriate challenge to the mouse, phenotypic differences might be revealed. This is in accordance with our previous work showing that *Hes3* null mice exhibit significantly altered responses to cuprizone-induced damage of oligodendrocytes in the brain and altered regeneration of oligodendrocytes afterwards^7^. It is possible that the roles of *Hes3* become more evident under conditions of challenge. In fact, this is very similar to what we observed in the pancreas, in our previous work: Hes3 null mice behave very similarly to WT mice, in terms of pancreatic function under normal conditions but exhibit much greater damage and reduced regeneration following streptozotocin-induced pancreas damage^11,12^. Therefore, future work may search for disease paradigms where the lack of *Hes3* results in clear brain phenotypes.

We report a number of mild phenotypes, some of which are observed under HFD conditions but not on ND conditions. For example, we found no differences in BW between *Hes3* null and WT mice when fed a ND, but significant differences when placed on a HFD. (In a separate cohort of mice placed under a distinct HFD protocol/composition, we did not observe statistically significant BW changes: Supplementary Material-GMC Report). Future studies may investigate the reasons behind this difference (fat tissue composition, thermoregulation, composition of diet, immune/inflammatory reactions, animal housing stresses, etc.).

Conversely, at the molecular level, we observed much more pronounced effects of the lack of *Hes3* in ND, compared to in HFD, regarding the expression of other members of the Hes/Hey gene family. It is possible that because in HFD *Hes3* expression is reduced relative to ND, the effect of lacking *Hes3* is not as pronounced. It is also possible that HFD regulates other Hes/Hey genes independently from *Hes3* in a manner that overrides, in part, the genetic lack of *Hes3*.

Here we show that STZ-induced β-cell damage, high fat diet, and metformin administration *in vivo* regulate *Hes3* levels in the adult mouse brain. Our data establish *Hes3* as a potentially valuable biomarker for the impact of diabetes on the plasticity potential of the brain. Because *Hes3* is a key component of a signal transduction pathway involved in neural stem cell biology and regeneration, it may also provide leads towards new therapeutic opportunities.

## Methods

All methods were performed in accordance with the relevant guidelines and regulations.

### Animals

6 week-old C57Bl6/J male mice were obtained from Janvier and they were used in accordance with the approved guidelines from the Landesdirektion Sachsen. The *Hes3* null mouse line was kindly provided by R. Kageyama^9^. At the GMC mice are housed according to the GMC housing conditions and German laws. All tests performed at the GMC were approved by the responsible authority of the district government of Upper Bavaria, Germany.

### Microscopy

Brains were dissected, fixed overnight and cryoprotected in 30% sucrose solution phosphate buffered saline at 4 °C until sinking. 16 μm brain tissue sections were prepared in the cryostat and mounted on glass slides. Immunofluorescence staining for *Hes3* was performed as described previously^11^. Images were acquired with a Zeiss LSM780 system. Image analysis was performed using the Fiji software.

### Tissue Collection

Brain tissues were dissected on ice. Depending on the experimental plan brain without cerebellum and olfactory bulb (indicated as BRAIN), brain without cerebellum, olfactory bulb and hypothalamus (indicated as Remaining Brain ‘’RB”), hypothalamus (HPT) and cerebellum (CBL) were dissected. We removed the olfactory bulbs for consistency as, sometimes, they are damaged during dissection. RB was split into two hemispheres and one was directly processed for RNA extraction. CBL was also divided in two parts and the same procedure was followed. Full HPT was used for the RNA extraction. Total RNA was isolated with the High Pure RNA isolation kit (Roche, 11828665001) and 1 μg of total RNA per sample was reverse transcribed using Promega M-MLV reverse transcriptase (Promega, M170B).

### Real-Time PCR

PCR was performed with DreamTaq Green DNA Polymerase (EP0712, ThermoScientific) with addition of betaine (5M, B0300, Sigma, B0300). qPCR experiments were performed with SsoFast EvaGreen Supermix (172–5201, Biorad) in a CFX384 Real time PCR Detection System^83^. Primer sets and reaction protocols are in Suppl. Tables 1–3. Relative gene expression was evaluated with the ΔΔCt method upon normalization to hypoxanthine-guanine phosphoribosyltransferase (HPRT). Primer sets and reaction protocols (Gene: Seq. 5′—> 3′ FW/Seq. 5′—> 3′ REV/Method) were:

mHes1: AAGATAGCTCCCGGCATTCCAAGC/AGCGCGGCGGTCATCTGC/PCR, qPCR;

m*Hes3*: AAAGCTGGAGAAGGCCGATA/TCCTTGCCTACGTCTCACCA/PCR;

m*Hes3*a: GTGATCTCCAAGCCTCTGATGGAGAA/CAGCTTTCGTTTCCGTATCTGATGTGA/PCR, qPCR;

m*Hes3*b: CCAGCAGCTTCCGAAAGATCTCCA/TCTCCAGCTTTCGTTTCCGTATCTGA/PCR, qPCR;

mHes5: CAACAGCAGCATAGAGCAGC/AGGCTTTGCTGTGTTTCAGG/PCR, qPCR;

mHes6: GGTGCAGGCCAAGCTAGAG/TGAAAGCTGCTACCCTCACG/PCR, qPCR;

mHes7: CCCAAGATGCTGAAGCCGTTGGT/AGCTTCGGGTTCCGGAGGTTCT/PCR, qPCR;

mHey1: AGGCATCATCGAGAAGCGCC/AGCTTAGCAGATCCCTGCTTCTCA/PCR, qPCR;

mHey2: TGAGAAGACTAGTGCCAACAGC/TGGGCATCAAAGTAGCCTTTA/PCR, qPCR;

mHeyL: CAGCCCTTCGCAGATGCAA/CCAATCGTCGCAATTCAGAAAG/PCR, qPCR;

mHPRT: AAGCTTGCTGGTGAAAAGGA/TTGCGCTCATCTTAGGCTTT/PCR, qPCR;

mMIF: TTAGCGGCACGAACGATCC/ACAGCAGCTTACTGTAGTTGC/qPCR.

The PCR protocol steps were (Cycling step: Temp, °C/Time/# of cycles): Initial Denaturation: 95/10 min/1; Denaturation: 95/4 min/35; Annealing: 60/30 sec/35; Extension: 72/1 min/35; Final Extension: 72/10 min/1.

The qPCR protocol steps were (Cycling step: Temp, °C/Time/# of cycles): Enzyme activation: 95/1 min/1; Denaturation: 95/5 sec/40; Annealing-Extension: 60/15 sec/40; Melt Curve: 65–95 °C in 0.5 °C increments/5 sec per step/1

### Aging

We examined 17 week old mice (from here on referred to as “Young”) and 34 week old mice (from here on referred to as “Old”).

### Animal models

#### Single High Dose STZ

8 week-old mice were injected intraperitoneally [phosphate-buffered saline (PBS) vehicle control or STZ (180 mg/kg, Sigma Aldrich, S0130)] and were euthanized 8 weeks later.

#### HFD

8 week old mice were fed Normal Diet (ND, 10% kcal % fat, D12450B, OpenSource Diets – Research Diets) or High Fat Diet (HFD, 60% kcal % fat, D12450B, OpenSource Diets – Research Diets) and euthanized after ~10 weeks (“Short Feeding”) or ~30 weeks (“Long Feeding”). For the HFD feedings performed at the GMC, please refer to the GMC Report in the Supplementary Materials section.

#### Metformin administration

Metformin was administered in the drinking water (2 g/l, Sigma, D-150959) of 8 week old mice for 8 weeks. Water was changed 2 times per week.

### Metabolic Analyses

***Body weight (BW)***: We measured the BW of the mice weekly at the same time of day. ***Intraperitoneal Glucose-Tolerance-Test (ipGTT****)*: Mice were used for the glucose tolerance test after a 16–18 hours-lasting overnight food-withdrawal. In the beginning of the test, the body weight of mice was determined. For the determination of the fasting blood glucose level, the tip of the tail was scored using a sterilized scalpel blade and a small drop of blood was analyzed with the Accu-Chek Aviva glucose analyzer (Roche/Mannheim). Thereafter mice were injected intraperitoneally with 2 g of glucose/kg body weight using a 20% glucose solution, a 25-gauge needle and a 1-ml syringe. 15, 30, 60 and 120 minutes after glucose injection, additional blood samples (one drop each) were collected and used to determine blood glucose levels as described before. Repeated bleeding was induced by removing the clot from the first incision and massaging the tail of the mouse. After the experiment was finished, mice were placed in a cage with plentiful supply of water and food. ***Fasting BW, Fasting Blood Insulin and Fasting Blood Glucose levels:*** Fasting BW and fasting blood glucose were measured with Accu-Chek glucose meter (Roche, Mannheim, Germany) and blood samples were collected for fasting insulin concentration determination with an Elisa Kit (Crystal Chem) and according to the manufacturer’s instructions.

### NSC cultures

#### Cell isolation

Fetal NSCs (fNSCs) were dissected and cultured from mouse embryos at embryonic day 13.5 (E13.5). Cells were grown in serum-free N2 medium; basic fibroblast growth factor [bFGF (233-FB-01M, R&D Systems)] was added to the cells at a concentration of 20 ng/ml daily^84^.

#### Treatments

Cells were seeded at 10,000 cells per well in a 12 well plate and treated with different concentrations of metformin (Sigma, D-150959) or exendin-4 [Ex-4 (Biotrend, BP0111)], beginning at 24 h after plating. Cells were fixed after 72 h with 4% paraformaldehyde (PFA) for 20 minutes and nuclei were stained with 4′,6-Diamidino-2 Phenylindole (DAPI).

#### Cell number, Proliferation

Cell proliferation was determined after 72 h by incubating the cells with 10 μM 5-ethynyl-2′-deoxyuridine (EdU) for 5 h and followed by visualization using the Click-IT EdU Alexa Fluor 594 Imaging Kit (Invitrogen C10339). Five images (using a 20x objective) from each well were acquired (3 wells per plate) with a standard Zeiss structured illumination microscope (Zeiss – Axio Observer Z1, inverted) and cells were counted using the Fiji software.

#### PCR/qPCR

For the time-course experiments cells were seeded at 500,000 cells per T25 flask and treatments (0 or 500 μM metformin) were initiated 24 h after plating. Cells were collected at 6, 24, 48 and 72 h and processed directly for PCR/qPCR experiments. Cell culture medium and treatments were changed daily.

### Mouse phenotyping

Comprehensive phenotypical characterization of *Hes3* null mice was performed at the German Mouse Clinic (GMC), Munich, Germany^85^. 64 mice (16 males, 16 females, 16 control males and 16 control females) were used beginning at age 9 weeks. Tests were conducted using the protocols described before^86^ and referenced at https://www.mouseclinic.de (Click on “VIEW RESULTS OF MUTANT LINES” or “phenomap” and search for project “*Hes3*_KO”). The methods described in Fig. S5 (for Behavior, neurology, and nociception phenotyping) as well as additional analyses can also be found in the Supplementary Material-GMC Report.

An additional cohort (11 males, 11 females, 11 control males and 11 control females) was fed a HFD (E15741-347 (D12492 mod.) Ssniff Spezialdiäten GmbH, Soest, Germany, containing 60 energy-% from beef tallow). Mice were subjected to the following tests beginning at age 12–13 weeks: Body composition analysis (qNMR, Minispec LF 50, Bruker, Ettlingen, Germany), 21 hours indirect calorimetry (Phenomaster, TSE Systems Gmbh, Bad Homburg, Germany), rectal body temperature, body surface temperature (thermosensor: Almemo ZA 9040, data logger: Almemo 2290-8, Ahlborn, Holzkirchen, Germany), and ipGTT after overnight food deprivation.

### Heat maps

Heat maps were generated in Morpheus https://software.broadinstitute.org/morpheus/.

### Statistical analyses

Data are expressed as means ± SEM. Statistical analyses were performed in Graphpad Prism (GraphPad Software, Inc., San Diego, CA). The Student’s t test, a Mann- Whitney U test, or one-way ANOVA were used and significance was set at p < 0.05. A detailed account of the statistical methods used in the phenotypic analysis of the *Hes3* null mice is provided in the GMC Report in the Supplementary Materials section.

### Data availability

The datasets generated during and/or analyzed during the current study are available from the corresponding author on reasonable request and in the https://www.mouseclinic.de repository (Click on “VIEW RESULTS OF MUTANT LINES” or “phenomap” and search for project “*Hes3*_KO”).

## Electronic supplementary material

Supplemental Figures
