# Supplementary material for: Streptozotocin-induced β-cell damage, high fat diet, and metformin administration regulate Hes3 expression in the adult mouse brain
Source: Sci Rep. 2018 Jul 27;8:11335. doi: 10.1038/s41598-018-29434-2 (PMC6063949; doi:10.1038/s41598-018-29434-2)
Supplement: Supplementary file 1 — Supplemental Figures [file 41598_2018_29434_MOESM1_ESM.pdf]

Supplementary Information

Streptozotocin-induced β-cell damage, high fat diet, and metformin administration regulate *Hes3* expression in the adult mouse brain

Author List

Polyxeni Nikolakopoulou[1], Antonios Chatzigeorgiou[2], Ioannis Kourtzelis[2], Louiza Toutouna[1], Jimmy Masjkur[1], Carina Arps-Forker[1], Steven W. Poser[1], Jan Rozman[3,4], Birgit Rathkolb[3,4,5], Juan Antonio Aguilar-Pimentel[3], German Mouse Clinic Consortium[§], Eckhard Wolf[5], Martin Klingenspor[6,7], Markus Ollert[8], Carsten Schmidt-Weber[9], Helmut Fuchs[3], Valerie Gailus-Durner[3], Martin Hrabe de Angelis[3,4,10], Vasiliki Tsata[11], Laura Sebastian Monasor[1], Maria Troullinaki[2], Anke Witt[2], Vivian Anastasiou[12], George Chrousos[13,14], Chun-Xia Yi[15,16], Cristina García-Cáceres[16], Matthias H. Tschöp[16,17], Stefan R. Bornstein[1], and Andreas Androutsellis-Theotokis*[1,11,18]

Contact Information

Andreas Androutsellis-Theotokis, Department of Medicine, University of Dresden, Fetscherstrasse 74, Dresden 01307, Germany, Tel: +49(0) 796-5690; Fax: +49(0) 351 458 6398; E-mail: andreas.theotokis@uniklinikum-dresden.de

Supplementary Figures and Legends

(Each Supplementary Figure is followed by its corresponding legend on the next page)

Figure S1

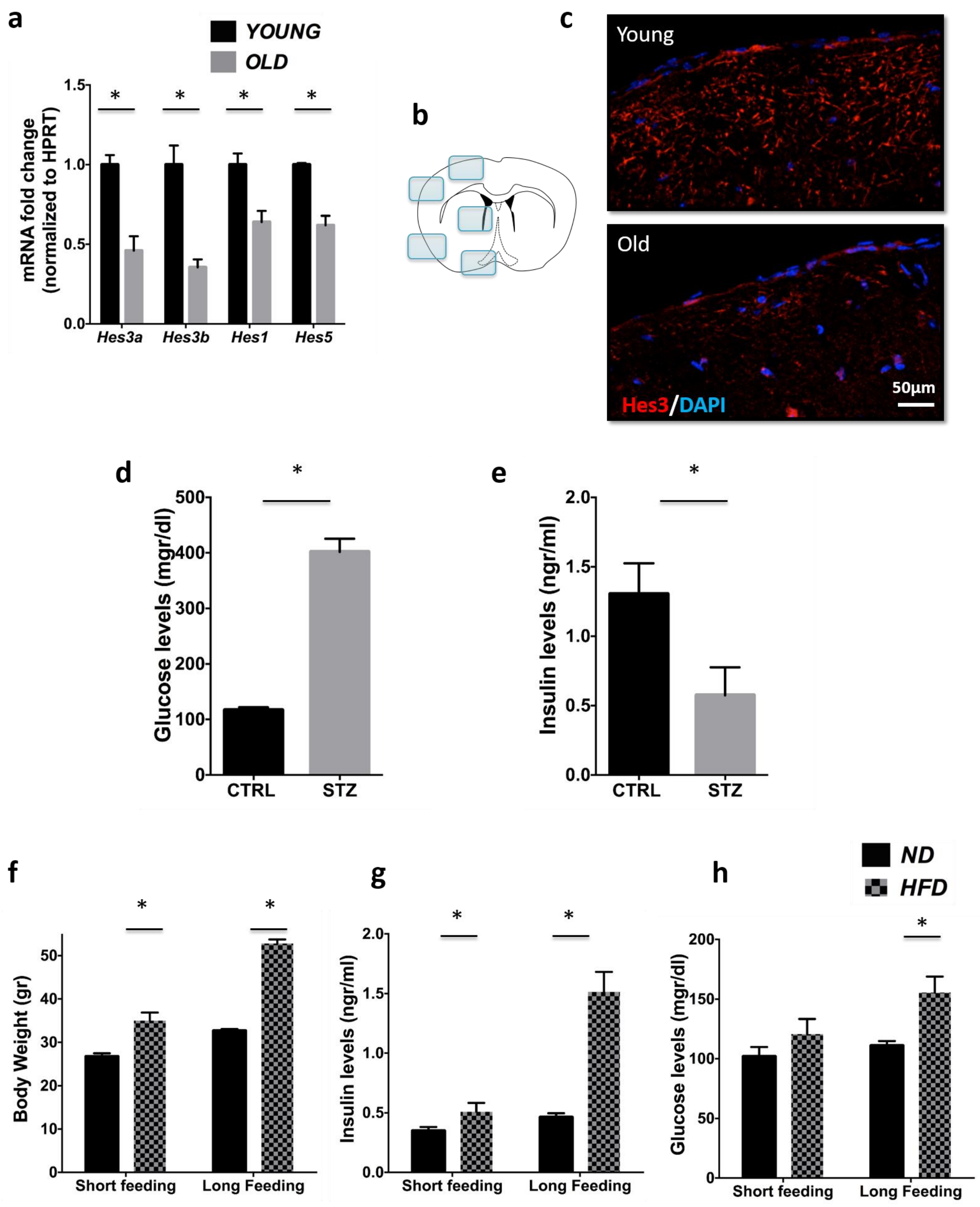
a
YOUNG
OLD
mRNA fold change (normalized to HPRT)
1.5
1.0
0.5
0.0
*
Hes3a
Hes3b
Hes1
Hes5
b
c
Young
Old
Hes3/DAPI
50µm
d
Glucose levels (mgr/dl)
500
400
300
200
100
0
CTRL
STZ
e
Insulin levels (ngr/ml)
2.0
1.5
1.0
0.5
0.0
CTRL
STZ
f
Body Weight (gr)
50
40
30
20
10
0
Short feeding
Long Feeding
g
Insulin levels (ngr/ml)
2.0
1.5
1.0
0.5
0.0
Short feeding
Long Feeding
h
ND
HFD
Glucose levels (mgr/dl)
200
150
100
50
0
Short feeding
Long Feeding

Figure S1. Aging, streptozotocin-induced β–cell damage and high fat diet regulate Hes3 expression in the brain.

(a) qPCR analysis of the relative mRNA expression of Hes/Hey genes in young and old mice (N=3-6). (b) Diagram showing the brain areas used for image acquisition and Hes3 signal measurement. (c) Examples of images used for Hes3 quantification. The particular images correspond to the top field marked in (b). Nuclei are stained with DAPI and are shown in blue. (d,e) A single high dose of STZ increases blood glucose levels and reduces insulin levels 8 weeks after injection, indicating the establishment of the STZ model (N=8; measurements were performed after an overnight fasting). (f-h) HFD increases BW, insulin, and glucose levels at different time points, indicating the establishment of the HFD model (N=4-10; measurements were performed after an overnight fasting). [Data are means ± SEM. Mann-Whitney test; $^*p<0.05$].

Figure S2

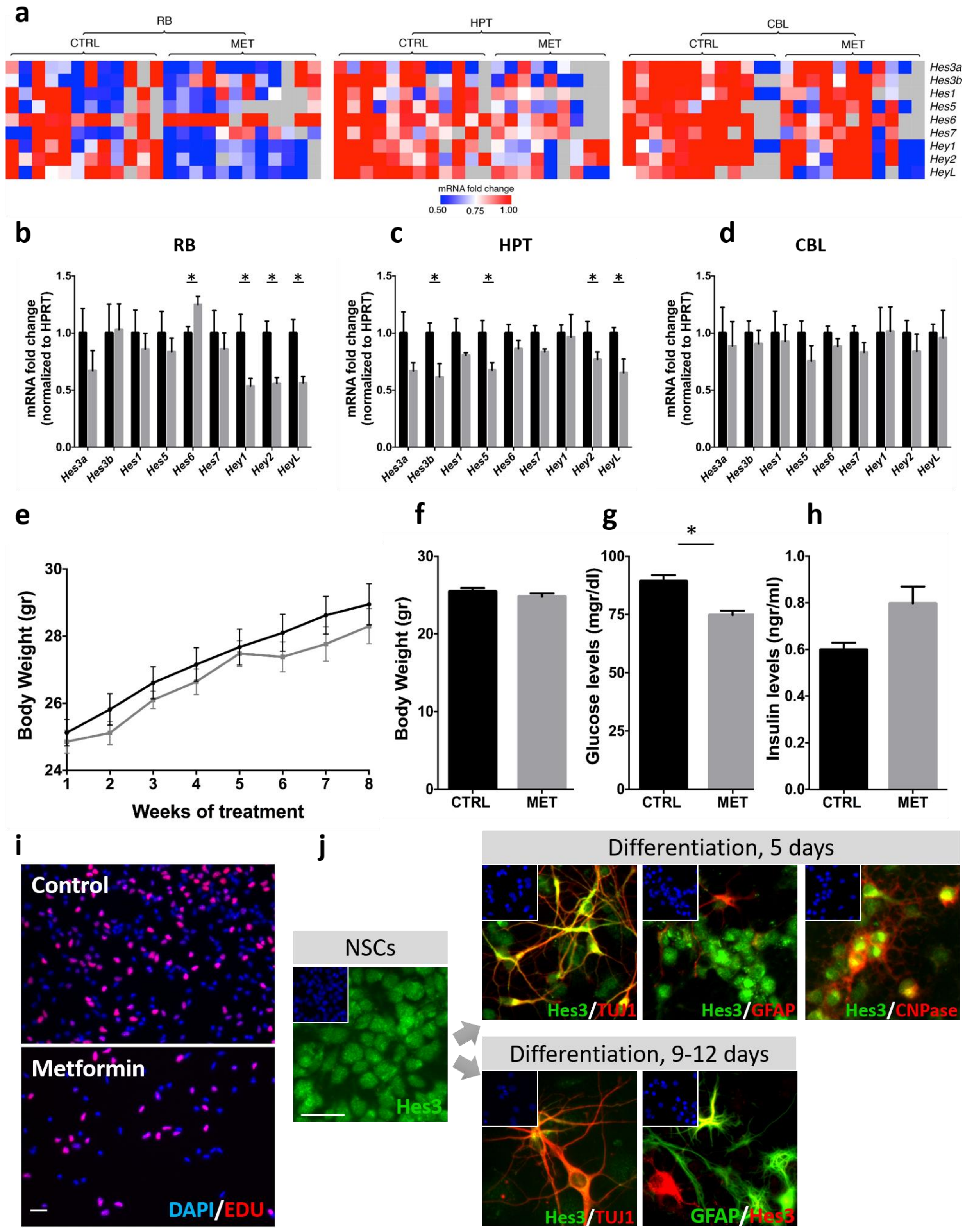
a
RB
CTRL
MET
HPT
CTRL
MET
CBL
CTRL
MET
Hes3a
Hes3b
Hes1
Hes5
Hes6
Hes7
Hey1
Hey2
HeyL
mRNA fold change
0.50
0.75
1.00
b
RB
c
HPT
d
CBL
mRNA fold change (normalized to HPRT)
Hes3a Hes3b Hes1 Hes5 Hes6 Hes7 Hey1 Hey2 HeyL
e
Body Weight (gr)
Weeks of treatment
f
Body Weight (gr)
CTRL
MET
g
Glucose levels (mgr/dl)
CTRL
MET
h
Insulin levels (ngr/ml)
CTRL
MET
i
Control
Metformin
DAPI/EDU
j
NSCs
Hes3
Differentiation, 5 days
Hes3/TUJ1
Hes3/GFAP
Hes3/CNPase
Differentiation, 9-12 days
Hes3/TUJ1
GFAP/Hes3

Figure S2. Metformin regulates Hes/Hey gene expression in vivo and in vitro.

(a) Heatmap showing gene expression levels of different Hes/Hey genes in different areas of the brain in control and metformin-treated mice. (N=5-12; MET, Metformin; the data are summarized per animal group and presented as a heat map in Figure 2a]. (b-d) Bar graph versions of the qPCR analysis shown in (a). (e) BW progression in mice treated with metformin (2g/l in the drinking water, continuously for 8 weeks), and control mice (normal drinking water; not fasted BW measurements; N=12). (f-h) BW, glucose, and insulin measurements in mice treated with metformin, and control mice. (N=12; measurements were performed after an overnight fasting). (i) Immunocytochemistry image examples of EdU incorporation 72 hours after treatment with metformin (500μM). EdU was added 5 hours before cell fixation. [Scale bar: 30 μm]. (j) Immunocytochemistry image examples of Hes3 expression in undifferentiated NSCs and following differentiation for different periods of time [Scale bar: 30 μm; Insets: DAPI. Note: Oligodendrocytes in the cell culture media do not survive for long periods of time]. [Data are means ± SEM. Mann-Whitney test; *p<0.05. HPRT was used as a reference gene].

Figure S3

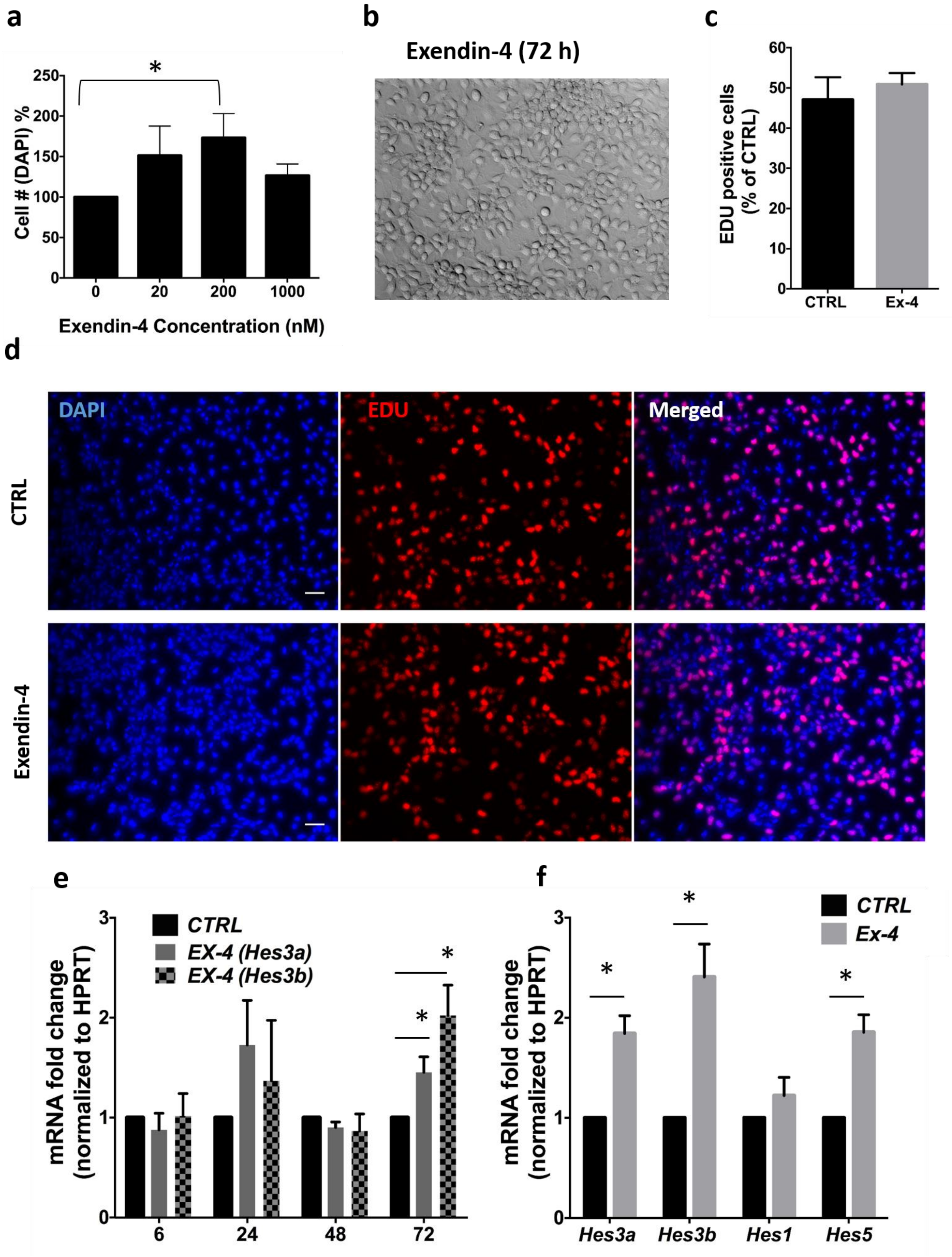
a
*
Cell # (DAPI) %
250
200
150
100
50
0
0
20
200
1000
Exendin-4 Concentration (nM)
b
Exendin-4 (72 h)
c
EDU positive cells (% of CTRL)
60
50
40
30
20
10
0
CTRL
Ex-4
d
DAPI
EDU
Merged
CTRL
Exendin-4
e
mRNA fold change (normalized to HPRT)
CTRL
EX-4 (Hes3a)
EX-4 (Hes3b)
3
2
1
0
6
24
48
72
f
CTRL
Ex-4
Hes3a
Hes3b
Hes1
Hes5

Figure S3. Exendin-4 regulates Hes/Hey gene expression in cultured mouse fNSCs. Related to Figure 2.

(a) Ex-4 increases cell number (DAPI-stained nuclei counts) in a dose-dependent manner (N=7; $p<0.05$ for the 72h Ex-4 dose of 200nM). (b) Brightfield image of primary mouse fNSC cultures treated with or Ex-4 (200nM) for 72 hours (to compare with Figure 2d). (c) Ex-4 (200nM) does not significantly alter EdU incorporation (N=3; 72h treatment). (d) Image examples of EdU incorporation 72 hours after treatment with Ex-4 (200nM). EdU was added 5 hours before cell fixation. [Scale bar: 30 µm]. (e,f) Ex-4 (200nM, 72h) regulates Hes/Hey gene mRNA levels in cultured fNSCs (N=3. Data are means ± SEM. Mann-Whitney test; *$p<0.05$. HPRT was used as a reference gene).

Figure S4

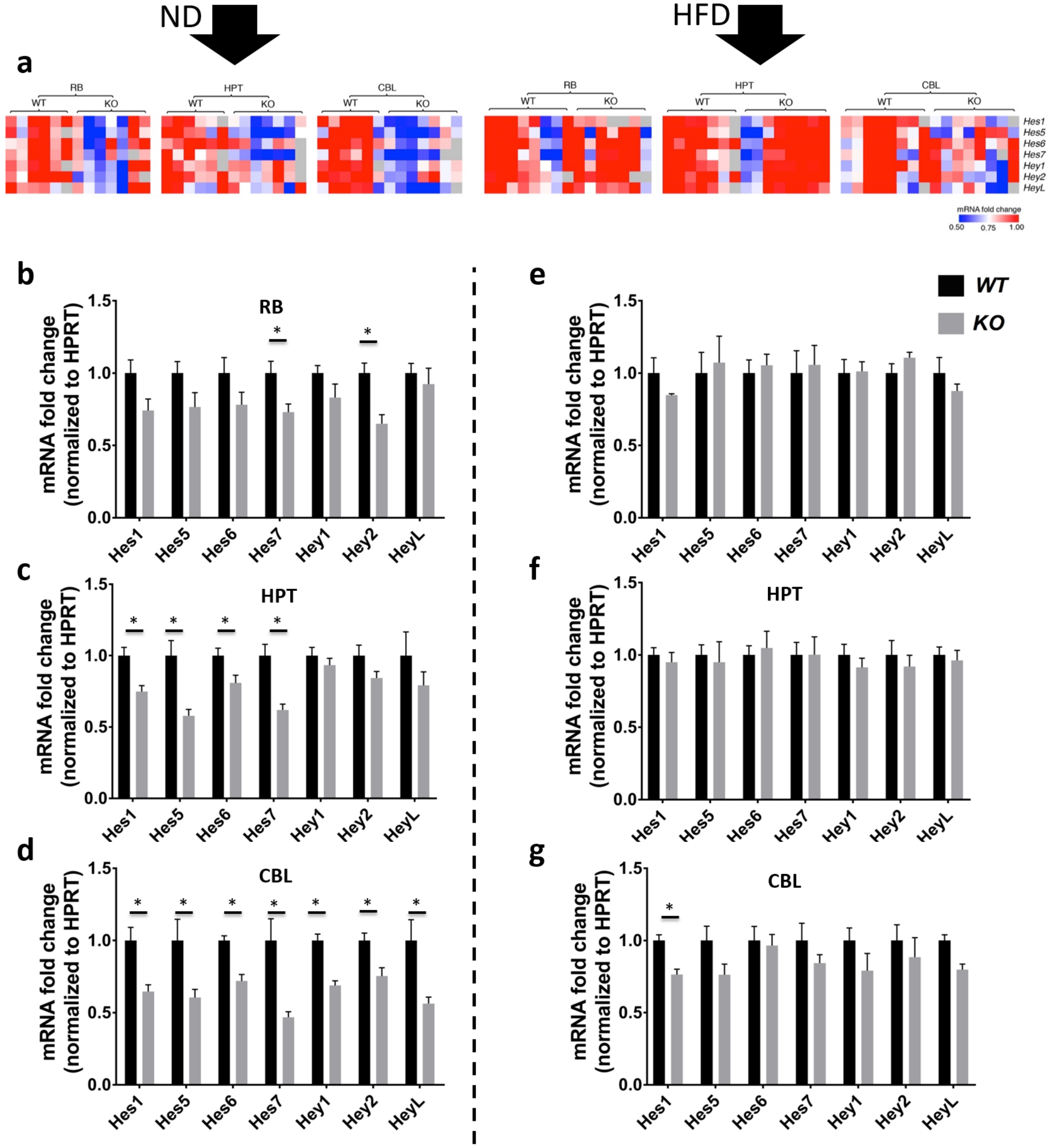

ND
HFD
a
RB
HPT
CBL
WT
KO
Hes1
Hes5
Hes6
Hes7
Hey1
Hey2
HeyL
mRNA fold change
0.50
0.75
1.00
b
c
d
e
f
g
WT
KO
mRNA fold change (normalized to HPRT)
1.5
1.0
0.5
0.0
*

Figure S4. Hes3 null mice exhibit altered expression of Hes/Hey genes in the brain.

(a) Heatmap showing gene expression levels of different Hes/Hey genes in different areas of the brain in WT and Hes3 null mice, in ND and HFD conditions. The heat map shows values from individual mice and corresponds to Figure 3a. (N=4-8). (b-g) Bar graph version of the qPCR analysis shown in (a). [Data are means ± SEM. Mann-Whitney test; $*p<0.05$. HPRT was used as a reference gene].

Figure S5

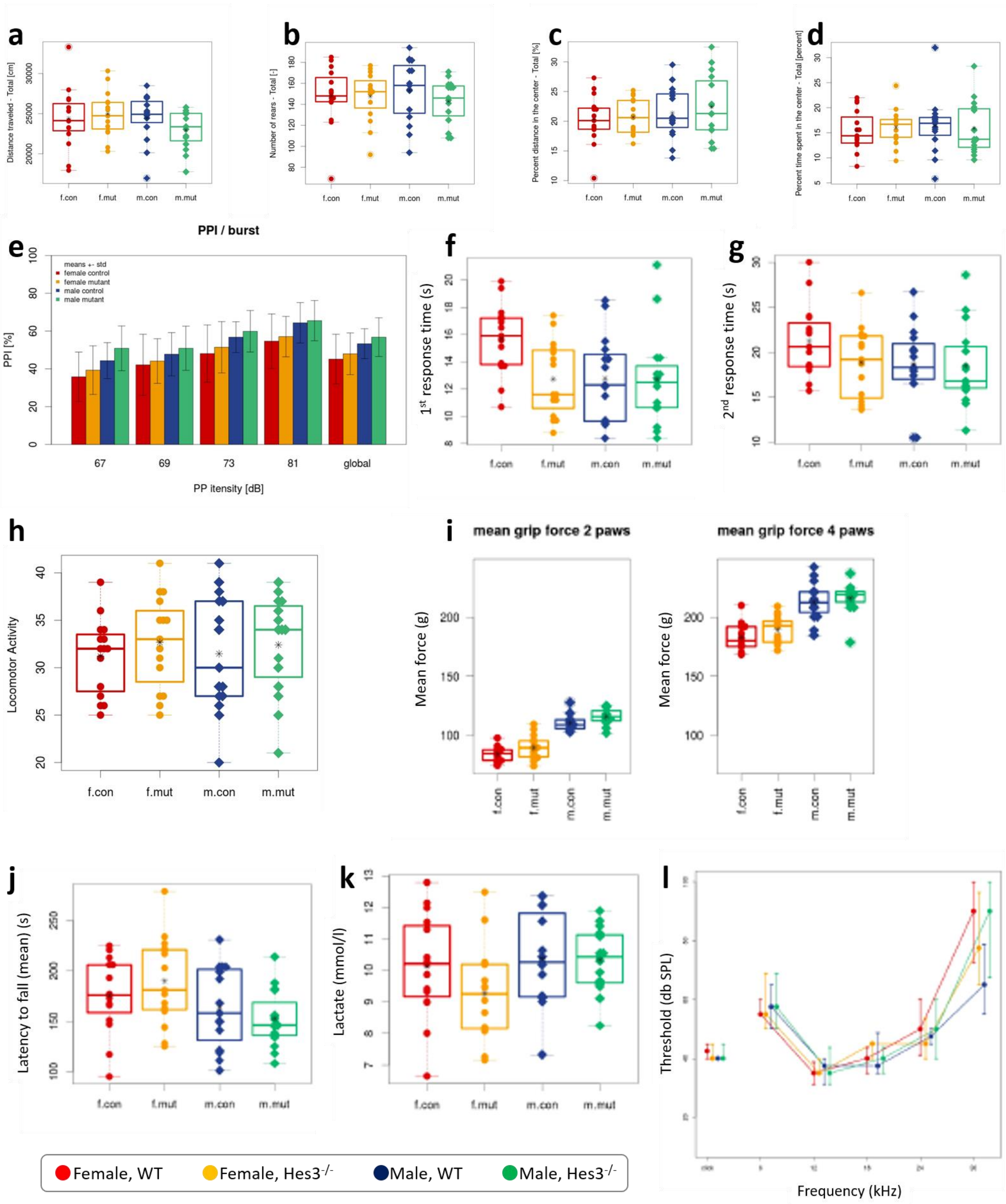

a
Distance traveled - Total [cm]
f.con
f.mut
m.con
m.mut
b
Number of rears - Total [-]
c
Percent distance in the center - Total [%]
d
Percent time spent in the center - Total [percent]
PPI / burst
e
means +- std
female control
female mutant
male control
male mutant
PPI [%]
67
69
73
81
global
PP itensity [dB]
f
1st response time (s)
g
2nd response time (s)
h
Locomotor Activity
i
mean grip force 2 paws
mean grip force 4 paws
Mean force (g)
j
Latency to fall (mean) (s)
k
Lactate (mmol/l)
l
Threshold (db SPL)
Frequency (kHz)
Female, WT
Female, Hes3-/-
Male, WT
Male, Hes3-/-

Figure S5. Behavior, neurology, and nociception phenotyping of Hes3 null mice (Selected data from https://www.mouseclinic.de). (a) Open field: Distance traveled - Total boxplot with stripchart, split by sex and genotype (b) Open Field: Number of rears - Total boxplot with stripchart, split by sex and genotype (c) Open Field: Percent distance in the center - Total boxplot with stripchart, split by sex and genotype (d) Open Field: Percent time spent in the center - Total boxplot with stripchart, split by sex and genotype (e) Prepulse Inhibition: PPI grouped barplot, split by sex and genotype (f) Hot Plate Test: 1st response time boxplot with stripchart, split by sex and genotype (g) Hot Plate Test: 2nd response time boxplot with stripchart, split by sex and genotype (h) SHIRPA: Locomotor Activity boxplot with stripchart, split by sex and genotype (i) Grip Strength: Mean force (2 and 4 paws) boxplot, split by sex and genotype (j) Rotarod: Latency to fall (mean) boxplot with stripchart, split by sex and genotype (k) Lactate: Boxplot with stripchart, split by sex and genotype (l) ABR: Stimulus intensity thresholds measured for different frequencies, medians and quartiles. Values above measurement limit (85db) are replaced by 100. [N numbers: a-k, 15; i: 10]

Figure S6

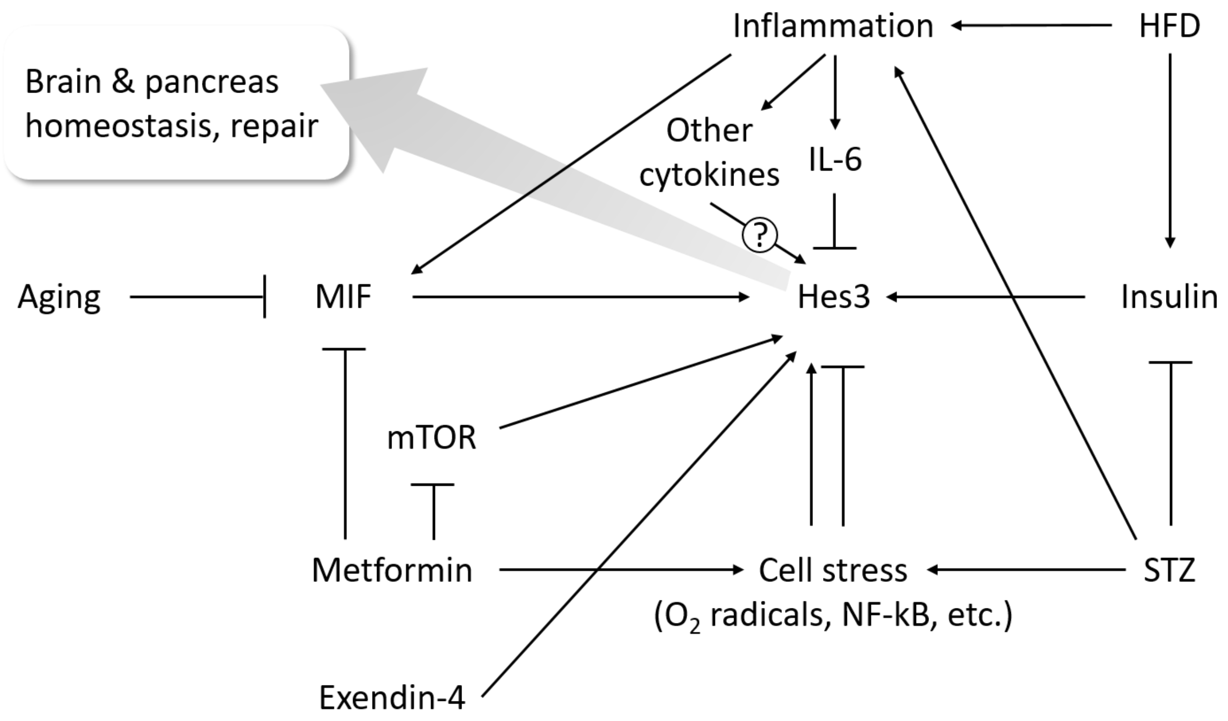
Inflammation
HFD
Brain & pancreas homeostasis, repair
Other cytokines
IL-6
?
Aging
MIF
Hes3
Insulin
mTOR
Metformin
Cell stress
(O_2 radicals, NF-kB, etc.)
STZ
Exendin-4

Figure S6. Conceptual diagram of how Hes3 in the brain may be integrating multiple biological parameters.

Aging opposes Hes3 expression in the brain. MIF is a positive regulator of Hes3 whose expression also drops with age. It is possible, therefore, that as MIF drops with age this contributes to the reduction of Hes3 expression we observed with age.

Insulin is another powerful inducer of Hes3. Therefore, in the STZ models, reduced circulating insulin levels may lead to a reduction in Hes3 levels in the brain. However, in the STZ models, cell stress caused by the damage to the organism may also promote an increase in brain Hes3 levels. Also, STZ-induced inflammation in the brain may further alter Hes3 expression. Which of these effects prevails may depend on the particular model used and the particular time point of the assessment of Hes3 levels.

HFD involves initial periods of inflammation and subsequent periods of prolonged elevated insulin. The effect of the HFD-induced inflammatory response on brain Hes3 may depend on the precise inflammatory response as some inflammatory cytokines such as the interleukins are predicted to oppose Hes3 expression (because they activate JAK which opposes Hes3 expression) whereas at least one other cytokine, MIF, has been demonstrated to increase Hes3 expression levels.

The type 2 diabetes medication metformin has multiple effects at the signal transduction level. One of the best described effects is that it opposes mTOR activity. Because mTOR is a potent activator of Hes3 expression, this result may explain our observation that metformin, *in vivo*, reduces Hes3 levels (in the hypothalamus of adult mice). Metformin may also induce stress effects on target cells, and this could lead to an increase in Hes3 expression. Our observations using cultured NSCs suggest this as a possibility, as we observed a reduction in cell growth, induced differentiation, and an increase in Hes3 expression.

Ex-4 induces Hes3 expression in cultured the cultured mouse insulinoma cell line MIN6. Similarly, Ex-4 increased Hes3 expression in cultured NSCs. It also increased cell number. Therefore, increased Hes3 expression can correlate with either increased cell number or decreased cell number. In the latter case, decreased cell number is accompanied by a differentiated morphology, suggesting that increased Hes3 expression may be a reaction to the onset of differentiation. Indeed, when NSC cultures are induced to differentiate by removal of mitogen in the medium, they also exhibit a transient increase in Hes3 expression.
